# Comparation of abdominal aortic balloon occlusion versus uterine artery embolization in the treatment of cesarean scar pregnancy

**DOI:** 10.3389/fmed.2024.1472239

**Published:** 2024-10-25

**Authors:** Tan Rui, He Wei, Jing Huaibo, Mou Han, Kenneth C. P. Cheung, Chai Yang

**Affiliations:** ^1^Department of Interventional Radiology, Chengdu Women's and Children's Central Hospital, School of Medicine, University of Electronic Science and Technology of China, Chengdu, China; ^2^Department of Radiology, Hospital of Chengdu Office of People's Government of Tibetan Autonomous Region, Chengdu, China; ^3^Hong Kong Phenome Research Center, Hong Kong Baptist University, Kowloon Tong, Hong Kong SAR, China

**Keywords:** cesarean scar pregnancy (CSP), abdominal aortic balloon occlusion, uterine artery embolization (UAE), uterine curettage, interventional therapy

## Abstract

**Study objective:**

This study is to uncover the advantages of abdominal aortic balloon occlusion in the uterine curettage treatment for patients with cesarean scar pregnancy (CSP).

**Methods:**

To retrospectively analyze the clinical data of eighty patients with CSP after treatment in our hospital from 01/10/2019 to 01/05/2021. The 80 patients were divided into 2 groups: 41 patients were treated with abdominal aortic balloon occlusion and the control group (*n* = 39) underwent Uterine artery embolization (UAE). The amount of bleeding during the operation, the operation time of the uterine curettage, the X-ray fluoroscopy time under DSA, the surface dose in radiation, the length of hospital stay (LOS), and the postoperative complications were compared between these 2 groups (type II and type III).

**Results:**

All the operations successfully retained the uterus. No balloon-related complications occurred in the experimental group. And in the control group, there were 14 cases of fever and 19 cases of pain after UAE. The fluoroscopy time of experimental group and control group were: (type II: (20.3 ± 7.1)s vs. (593.7 ± 284.5)s, *p* < 0.01), (type III: (21.2 ± 7.2)s vs. (509.8 ± 164.2)s, *p* < 0.01), the surface dose in radiation: (type II: (1.9 ± 0.7)mGy vs. (248.3 ± 85.9)mGy, *p* < 0.01), (type III: (2.1 ± 0.8)mGy vs.(252.0 ± 74.9)mGy, *p* < 0.01), the amount of bleeding during the operation: (type II:30.0(20.0, 50.0)ml vs. 20.0(10.0, 50.0)ml, *p* = 0.113), (type III:50.0 (17.5,162.5)ml vs. 50.0 (22.5, 72.5)ml, *p* = 0.623), the operation time of the uterine curettage:(type II: (54.8 ± 19.4)min vs.(43.9 ± 21.9)min, *p* = 0.071), (type III: (65.2 ± 50.4)min vs.(52.8 ± 20.1)min, *p* = 0.426), LOS: (type II:(5.4 ± 1.7)d vs.(5.4 ± 1.2)d, *p* = 0.816), (type III:(5.8 ± 2.4)d vs. (7.0 ± 1.7)d, *p* = 0.161). The follow-up was more than 3 months. No adverse reaction in the experimental group and 6 patients in the control group presented menstrual volume decrease.

**Conclusion:**

No balloon-related complications occurred in the abdominal aortic balloon occlusion and lower radiation exposure for both the operator and patient. And both abdominal aortic balloon occlusion and UAE can effectively reduce the bleeding during uterine curettage in patients with type II and III CSP.

## Background

**Figure 1:**
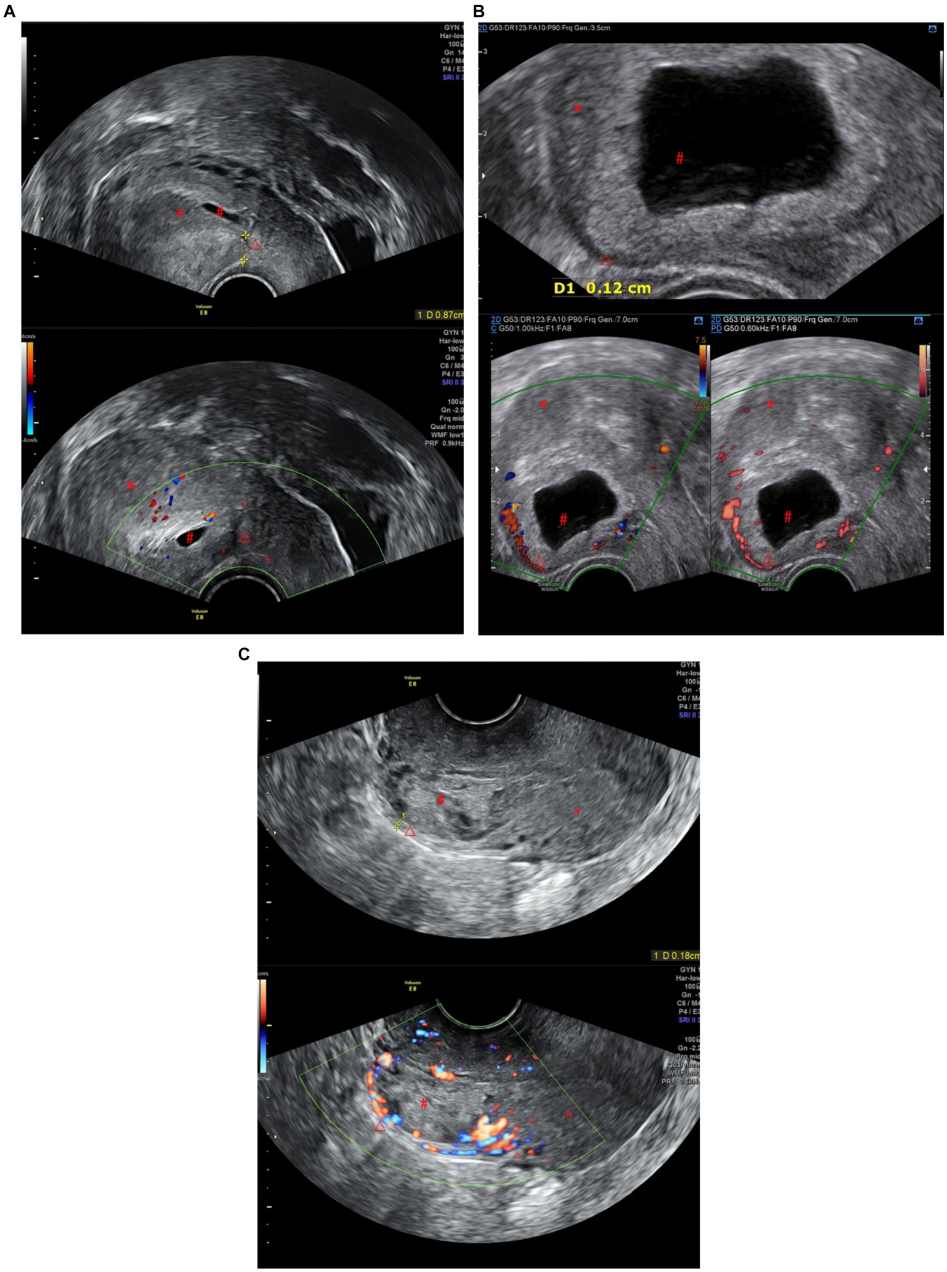
(A) CSP Type I: The gestational sac is partially located within the incision scar, the scar thickness is >3 mm, and there are dot-line blood flow signals around it. (B) CSP Type II: The gestational sac is partially located within the incision scar, the scar thickness is less than 3 mm, and there is a ring-shaped blood flow signal around it. (C) CSP Type III: The gestational sac is completely located within the incision scar, the scar thickness is less than 3 mm, there are abundant blood flow signals around it, and it protrudes locally outward (^*^Uterus, ^#^gestational sac, ^△^Cesarean Scar).

Cesarean scar pregnancy (CSP) is one type of serious ectopic pregnancy that occurs when the fertilized egg implants in the scar tissue after cesarean section. Immediate uterine curettage could avoid the risk of uterine rupture, hemorrhage, and placental implantation (1). Due to the thin muscular layer, rich blood flow, and weak or no contraction ability of cesarean scar site, it is easy to cause massive hemorrhage and even uterine perforation during the operation, and even be a serious threat to the patient safety. The increasing rate of cesarean sections has contributed to a rise in the significant incidence of CSP (2). It is typically divided into three types based on the location of the gestational sac at the scar and the thickness of the myometrium between the sac and the bladder (3) (Figure 1). Patients with type I CSP had a low risk of bleeding and could be treated with general methotrexate or routine abortion, so they were not included in this study. The other two types of CSP are defined as follows: Type II: (1) the gestational sac is partially deposited in the uterine scar; (2) the gestational sac is deformed; (3) the thickness of the myometrium between the gestational sac and the bladder is ≤3 mm; (4) CDFI: trophoblastic blood flow signal is seen in the scar (low obstruction of blood flow). Type III: (1) the gestational sac is completely embedded in the myometrium at the uterine scar and convex toward the bladder; (2) the uterine cavity and the cervical canal are empty; (3) the myometrium between the gestational sac and the bladder is obviously thinned or even missing, and the thickness of the myometrium is ≤3 mm; (4) CDFI: trophoblastic blood flow signals are seen at the scar (low obstruction of blood flow). The muscular thickness at the scar in the anterior uterine wall of types II and III CSP is ≤3 mm in both cases, with a rich blood supply at the scar, and the risks of major bleeding and residual pregnancy tissues are higher. As such, pursuing safe and effective treatment options for this condition is critical. While uterine artery embolization (UAE) is commonly used as a pretreatment for type II and type III CSP before uterine curettage to reduce the risk of bleeding during uterine curettage or CSP pregnancy removal surgery, and also greatly reduce the hysterectomy rate (3), it could result in postoperative complications, such as oligomenorrhea and even amenorrhea, and intrauterine adhesion. To explore potential alternatives, this study aims to evaluate the safety and effectiveness of abdominal aortic balloon occlusion for the treatment of CSP patients. Such a strategy may prove to be a viable solution that could reduce the risk of complications and improve outcomes for those with CSP.

## Materials and methods

### General data

This study was performed in accordance with the Declaration of Helsinki and approved by Chengdu Women’s and Children’s Central Hospital Ethics Committee which waived the requirement for informed consent. A total of 80 patients were observed for CSP during their hospitalization in our center from 01/10/2019 to 01/05/2021. To determine the type of CSP, all patients underwent diagnostic assessments based on the growth pattern of the gestational sac at the uterine anterior wall scar and the thickness of the muscular layer at the uterine scar. All patients were diagnosed as type II or type III CSP (3, 4). The patients were given a detailed explanation of the methods, advantages, and disadvantages of UAE and abdominal aortic balloon occlusion before making their choice. According to the choice of patients, a total of 41 patients (23 cases of type II and 18 cases of type III) were enrolled in the experimental group, while 39 patients (27 cases of type II and 12 cases of type III) were enrolled in the control group. The mean age of the experimental group was (33.3 ± 4.7) years old, and the mean age of the control group was (32.8 ± 3.8) years old. The mean gestational age was (48.7 ± 10.1) days for the experimental group and (46.8 ± 9.7) days for the control group. The maximum diameter of the gestational sac was (3.0 ± 1.6) cm for the experimental group and (2.9 ± 1.5) cm for the control group. There was no statistically significant difference between the two groups (all *p >* 0.05) (Table 1); both groups had a history of one or more cesarean sections.

**Table 1:** Comparation of basic data between the two groups.

Groups	Number of cases	Age	Gestational age (d)	Maximum diameter of gestational sac (cm)
Experimental group	41	33.3 ± 4.7	48.7 ± 10.1	3.0 ± 1.6
Control group	39	32.8 ± 3.8	46.9 ± 9.7	2.9 ± 1.5
*t* value		0.601	0.816	0.534
*p* value		0.550	0.417	0.595

### Surgical methods

**Figure 2:**
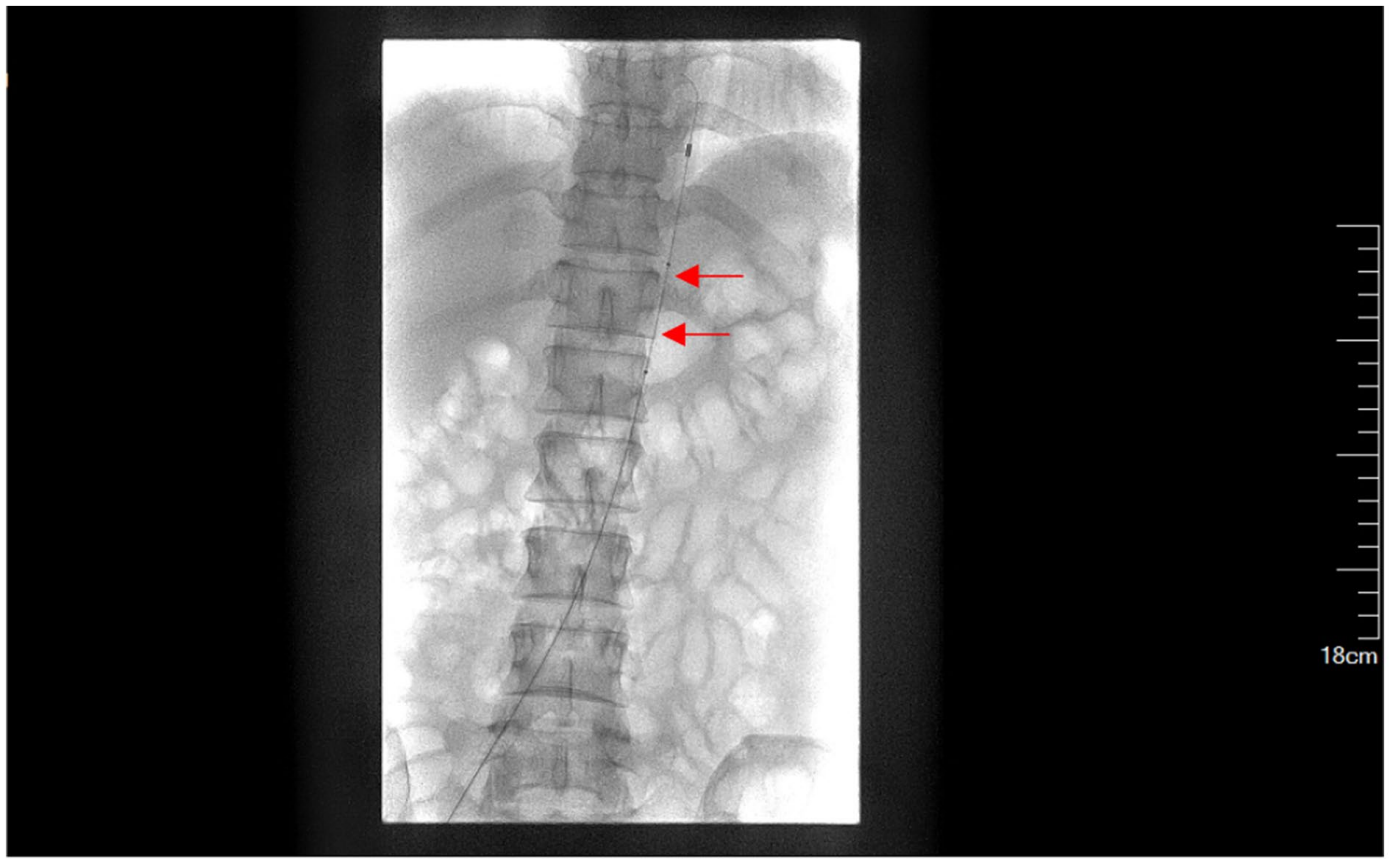
Presetting of abdominal aortic balloon (a red arrow indicates the balloon labeled at T12-L1).

In the experimental group, the abdominal aorta balloon catheter was preset under digital subtraction angiography (DSA) before uterine curettage. Femoral artery puncture was performed under local anesthesia, and 8 F sheath tube was inserted. An 18 mm × 40 mm balloon catheter (BARD Medical Products, USA) was then inserted through the sheath tube into the abdominal aorta at the thoracic 12-lumbar 1 level (Figure 2) (5). During the uterine curettage with hysteroscopy or combined hysteroscopy and laparoscopy, the abdominal aorta was intermittently blocked (every 15 min for 1–2 min), and the significant reduction of bleeding or no bleeding in the surgical field was considered as effective occlusion. The embryo was removed, and the bleeding point was electrocoagulated or suture ligated to stop bleeding. Excision of the gestational lesion and suture of the lesion was performed when necessary. At the end of the operation, the balloon was withdrawn, and the femoral artery puncture point was bandaged.

**Figure 3:**
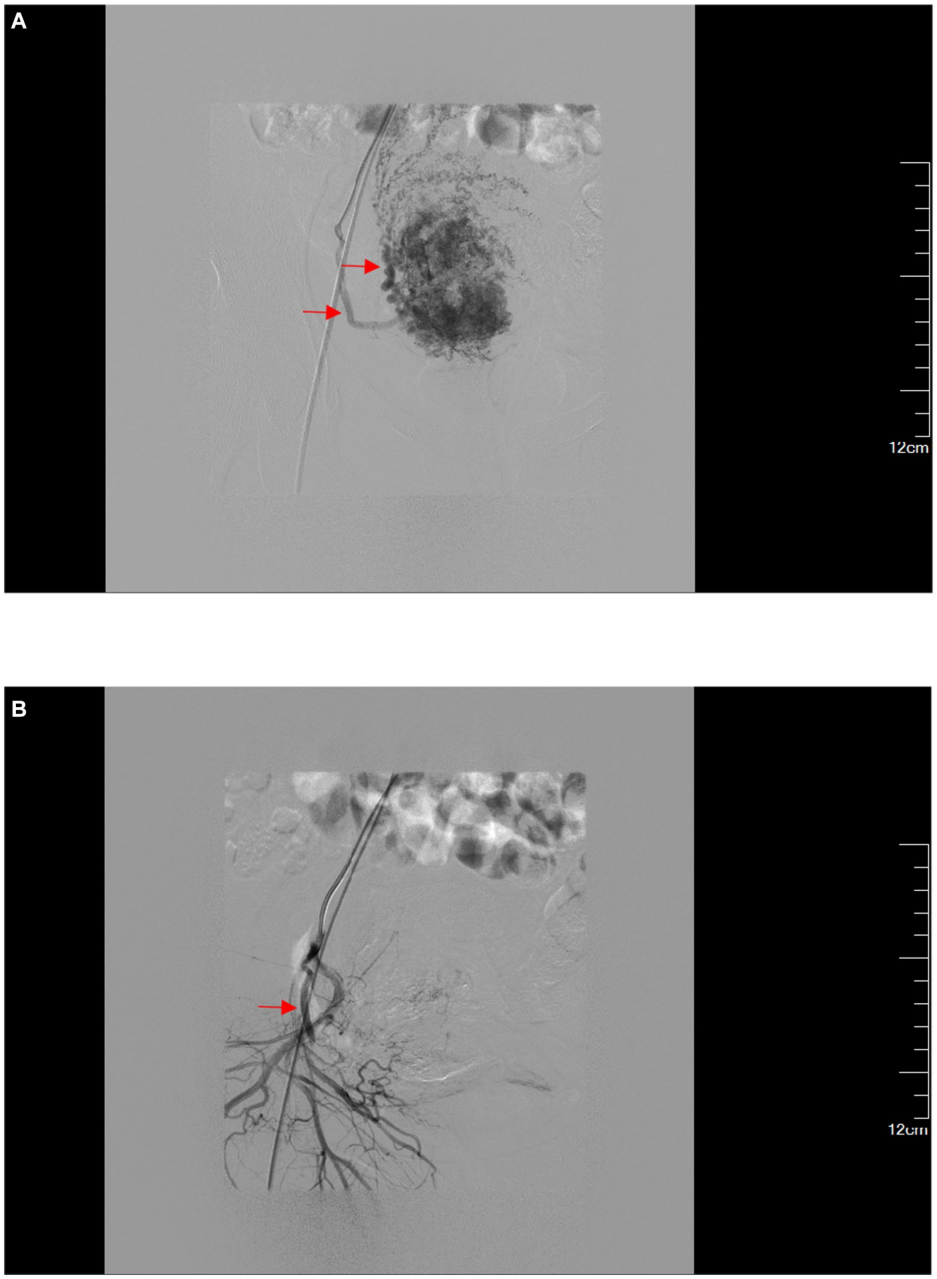
(A) Superselective angiography of the right uterine artery. (B) After embolization of the right uterine artery, the trunk is developed while the distal terminals are not developed.

In the control group, UAE was performed under local anesthesia, and 1,000 μm gelatin sponge particles (Products of Hangzhou Alikang Medical Technology Co., LTD.) were used to embolize the uterine artery terminal vessels without visualization (Figure 3). The uterine curettage with hysteroscopy or combined hysteroscopy and laparoscopy were performed on the same day.

### Outcome measures and follow-up

The main observation indicators for both the experimental group and the control group were the amount of bleeding during the operation (evaluated by gynecological surgeons after the operation), the operation time of the uterine curettage, the X-ray fluoroscopy time under DSA, the surface dose in radiation, the length of hospital stay, and any postoperative complications. Patients were followed up for over 3 months to observe potential reduction in menstrual volume or intrauterine adhesion.

### Statistical analysis

SPSS 24.0 software was used for statistical analysis. Normally distributed measurement data were presented as *xˉ* ± *s* and compared using *t*-tests. Non-normally distributed measurement data were presented as *M* (P25, P75) and compared using the Mann–Whitney *U* test. Count data were presented as rates and analyzed using the *χ*2 test or Fisher’s exact test. *p* values less than 0.05 were considered statistically significant.

## Results

### Comparison of the general data of the patients

There were no significant differences among the 2 groups in mean age, mean gestational age and maximum diameter of the gestational sac (all *p* > 0.05, Table 1).

### Comparation of the outcome measures with different surgical methods in patients with type II and III CSP

In the experimental group, 41 patients with CSP underwent successful implantation of an abdominal aortic balloon and were assisted with uterine curettage. During the operation, the uterine body color changed from red to pale due to the significant and effective effect of balloon occlusion of blood flow. There was no obvious bleeding or only a small amount of bleeding in the surgical field, and the bleeding was stopped successfully by electrocoagulation or suture. The balloon and sheath tube were safely removed after the operation. In the experimental group, only one case of vaginal hemorrhage occurred after uterine curettage, which was treated with emergency UAE and stopped the bleeding. No complications like balloon injury, thrombosis, or lower limb and pelvic organ ischemia–reperfusion injury observed in the experimental group.

In the control group, 39 patients with CSP underwent UAE and uterine curettage successfully. During the operation, pale ischemia changes of varying degrees were observed in the uterine body, and the amount of bleeding during the operation was less. Both groups had succeeded in completing the operation with preservation of the uterus. In the experimental group, type II and type III CSP patients were compared to those in the control group separately. For type II, there was no significant difference in intraoperative bleeding or LOS between the two groups (*p* > 0.05). However, the experimental group had less X-ray fluoroscopy time and surface radiation dose under DSA, but a longer operation time than the control group, with significant statistical differences (*p* < 0.01). For type III, there was no significant difference in intraoperative blood loss, operation time, and length of hospital stay between the two groups (*p* > 0.05). The experimental group displayed less X-ray fluoroscopy time and surface radiation dose under DSA than the control group, with significant statistical differences (*p* < 0.01) (Table 2).

**Table 2:** Comparation of treatment between the two groups.

Items	Type	Observation group	Control group	*t*/*z* values	*p* value
Fluoroscopy time (s)	II	20.3 ± 7.1	593.7 ± 284.5	−9.647	< 0.01
	III	21.2 ± 7.2	509.8 ± 164.2	−12.721	< 0.01
Body surface radiation dose (mGy)	II	1.9 ± 0.7	248.3 ± 85.9	−13.739	< 0.01
	III	2.1 ± 0.8	252.0 ± 74.9	−14.287	< 0.01
Blood loss during curettage (ml)	II	30.0 (20.0, 50.0)	20.0 (10.0, 50.0)	−1.584	0.113
	III	50.0 (17.5, 162.5)	50.0 (22.5, 72.5)	−0.492	0.623
Operation time of curettage (min)	II	54.8 ± 19.4	43.9 ± 21.9	1.847	0.071
	III	65.2 ± 50.4	52.8 ± 20.1	0.808	0.426
LOS (d)	II	5.4 ± 1.7	5.4 ± 1.2	−0.234	0.816
	III	5.8 ± 2.4	7.0 ± 1.7	−1.439	0.161

In the observation group, there were 23 cases of type II and 18 cases of type III. Control group: 27 cases of type II, 12 cases of type III.

In the control group, 14 cases experienced varying degrees of fever, while 19 cases reported pain after the embolization procedure. However, in the experimental group, there were no postoperative reactions observed. All patients from both groups were followed up for a period of more than 3 months and there were no incidences of uterine bleeding, amenorrhea, or intrauterine adhesion. Additionally, no adverse reactions occurred in the experimental group. As for the control group, 6 patients or 22.2% experienced a decrease in menstrual volume (Table 3).

**Table 3:** Comparation of postoperative conditions between the two groups.

Type	Groups	Abdominal pain		Fever		Decreased menstruation	
		Number of cases	Percentage (%)	Number of cases	Percentage (%)	Number of cases	Percentage (%)
II	Experimental group (*n* = 23)	0	0	0	0	0	0
	Control group (*n* = 27)	15	55.6	10	37.0	6	22.2
*p*^a^ value		< 0.01		< 0.01		0.025	
III	Experimental group (*n* = 18)	0	0	0	0	0	0
	Control group (*n* = 12)	4	33.3	4	33.3	0	0
*p*^a^ value		< 0.01		< 0.01		/	

^aDenotes Fisher’s exact probability method.^

## Discussion

In recent years, the global rate of cesarean section has been steadily increasing due to various social and family-related reasons. In 2000, the rate of cesarean section accounted for approximately 12.1% of all deliveries worldwide, which then rose to 21.1% in a span of 5 years. As the use of cesarean section continues to increase, so does the prevalence of cesarean scar pregnancy (CSP) (2). CSP is usually associated with serious complications: fetal death, prematurity, PAS, uterine rupture, severe hemorrhage, need for hys- terectomy and maternal death. Silva B et al. (6) reported that among 194 patients with CSP who were treated expectantly, 54 (27.8%) had severe maternal bleeding, 26 (13.4%) had uterine rupture, 9 (4.6%) suffered surgical complications and there was 1 maternal death, and ultimately 102 (52.6%) patients had a hysterectomy (10 elective versus 92 emergency). The diagnosis of CSP is primarily dependent on ultrasound and MRI. It is typically divided into three types based on the location of the gestational sac at the scar and the thickness of the myometrium between the sac and the bladder (3). In type II and type III, the myometrium between the gestational sac and the bladder is thinner, measuring ≤3 mm, and the local blood flow is often rich, sometimes causing the formation of a localized mass. Lack of contraction may easily result in extensive bleeding, uterine perforation, and even death during uterine curettage. These can inflict significant physical and psychological harm on patients. There are numerous treatment options for CSP and no international standardization. Commonly used treatments depending on the type of CSP include, but are not limited to, methotrexate injections, clearing surgery, pregnancy removal and uterine scar repair, hysterectomy, uterine artery embolization, and abdominal aortic balloon occlusion (3, 7–9).

The expert consensus (3) suggests that UAE is a suitable treatment option for patients with type II and type III CSP who require immediate hemostasis due to massive bleeding resulting from CSP surgery or spontaneous abortion. UAE can reduce the risk of bleeding during uterine curettage or CSP pregnancy removal, as type II and type III CSP are associated with rich blood supply prior to surgery. UAE is a minimally invasive procedure that can significantly reduce bleeding during uterine curettage of CSP, decrease the hysterectomy rate, and promote faster postoperative recovery (10). However, it should be noted that the embolic agent used in UAE is an absorbable material, and the uterus experiences a prolonged reduction in blood supply prior to full recovery. This can result in endometrial growth restriction and potential ischemic tissue infection, leading to postoperative fever. If the degree of embolization is too strong or the size of the embolic agent is not appropriate, delayed absorption may lead to prolonged uterine artery ischemia, ischemic necrosis of uterine basal cells, and severely reduced ability of endometrial regeneration and recovery. In such cases, endometrial atrophy, intrauterine inflammatory tissue adhesion, and even permanent uterine amenorrhea may occur (11, 12). Pain is another common post-embolization syndrome following UAE, resulting from uterine ischemia, hypoxia, congestion, edema, and local aseptic inflammatory reaction. This can cause limited activity and increased tension and anxiety in the patients. Moreover, UAE involves the use of a catheter for superselective embolization and angiography to assess the degree of embolization, increasing the X-ray fluoroscopy time and radiation dose to the patient. Furthermore, some studies (13) have found that excessive pressure from the embolic agent can cause it to enter the ovarian artery through a communication branch shared between the uterine and ovarian arteries, potentially affecting ovarian function. However, some research data (14) suggest that uterine artery embolization has little or no effect on ovarian function in women under 40 years of age. This may require further research.

Abdominal aortic balloon occlusion has become increasingly popular in recent years for cesarean section of obstetric pernicious placenta previa. Compared with other interventional techniques, this approach only requires unilateral puncture and does not require precise superselection of vessels. It significantly reduces the fluoroscopy time, fetal radiation dose, and interventional embolization complications, effectively controlling bleeding (15, 16). This study involves placing the abdominal aortic balloon in the abdominal aorta of the thoracic 12-lumbar 1 level, which is simple and takes only a few seconds of X-ray fluoroscopy time, resulting in a much lower radiation dose to the operator and patient compared to UAE. Additionally, risks of post-embolization syndrome and uterine or ovarian amenorrhea caused by UAE are avoided. During uterine curettage in 41 patients with type II and III CSP treated with abdominal aortic balloon occlusion, intermittent occlusion of the abdominal aorta (every 15 min for 1–2 min) and hysteroscopy or hysteroscopy combined with laparoscopy were used to locate the bleeding site. After electric coagulation or suture hemostasis was completed, the balloon catheter and vascular sheath were removed. Except for one case of postoperative bleeding and UAE, the others were successfully treated, and no complications related to balloon operation occurred. The results are consistent (9) with previous studies, suggesting that abdominal aortic balloon occlusion is effective in reducing intraoperative bleeding and has lower risk and fewer complications for patients with type II and III CSP. However, abdominal aortic balloon obstruction is an invasive endovascular operation, which may cause damage to the endothelium and the abdominal aorta during the operation, triggering serious complications such as balloon catheter migration and organ ischemic necrosis (17). Study (18) shows that single balloon occlusion within 60 min is safe. Although complications of abdominal aortic balloon obstruction are rare, it is still important to perform a good preoperative evaluation and gentle intraoperative maneuvers to avoid more harm to the patient.

This study suggests that abdominal aortic balloon occlusion can assist in uterine curettage for patients with type II and III CSP. This technique effectively controls bleeding during the procedure, avoiding the need for hysterectomy. Additionally, it shortens X-ray fluoroscopy time and reduces radiation dose for both operator and patient, without affecting blood supply to the uterus and ovaries. Compared to UAE, it also avoids the risk of complications such as uterine or ovarian amenorrhea. For preventing the risk of uterine hemorrhage, abdominal aortic balloon occlusion is a better option than UAE. However, the sample size needs to be expanded, and a better implementation scheme must be explored. It is worth noting that abdominal aortic occlusion requires cooperation from an interventionist.

## Data Availability

The original contributions presented in the study are included in the article/supplementary material, further inquiries can be directed to the corresponding authors.
